# New Pharmacotherapeutic Classes for the Management of Heart Failure: A Narrative Review

**DOI:** 10.7759/cureus.57180

**Published:** 2024-03-29

**Authors:** Abhishek Haryani, Arushi Sangwan

**Affiliations:** 1 Medicine, Mahatma Gandhi Memorial Medical College, Indore, IND; 2 Radiology, Paras Hospital, Panchkula, IND

**Keywords:** heart failure, ularitide, omecamtiv mecarbil, vericiguat, heart failure drugs, management of heart failure

## Abstract

Heart failure (HF) is a syndrome characterized by the heart failing to pump blood to the body at a rate proportional to its needs. HF is a public health burden globally and one of the leading causes of hospitalizations in adults. While many classes of drugs have been introduced for the treatment of HF, not every drug may be well-tolerated by patients. In this narrative review, we describe a few of the newer classes of medications proposed to be efficacious in treating acute and chronic HF. We focus on vericiguat, omecamtiv mecarbil, ularitide, and serelaxin, and thoroughly examine their efficacy and safety profiles while summarizing the clinical trials of the drugs. There is a need for more long-term studies comparing the efficacy of these medications to the conventional ones.

## Introduction and background

The burden of heart failure (HF) on public health globally has been growing exponentially. Recent epidemiological studies show that 64.34 million people suffer from HF globally and it accounts for 9.11 million years lost due to disability (YLD) [1].

HF has a diverse etiology, and it eventually leads to structural and/or functional changes in the heart. The American College of Cardiology (ACC) and American Heart Association (AHA) classify HF into four stages based on the progression of the disease: stage A - at risk for HF; stage B - pre-HF; stage C - symptomatic HF; and stage D - advanced HF [2].

Current guidelines suggest a stepwise approach to the treatment of HF based on the progression of the disease. Lifestyle changes such as salt and fluid restriction, physical exercise, and weight loss are the first step, followed by the pharmacological treatment, which includes a wide array of medications, including angiotensin-converting enzyme inhibitors (ACEi), angiotensin receptor blockers (ARB), other diuretics, beta-blockers, inotropic drugs, and vasopressin receptor antagonists. While a large variety of medications for the treatment of HF exist, not all of them are effective and/or safe for patients with different co-morbidities.

In this narrative review, we aim to describe a few of the new classes of medications being researched for the treatment of HF. As some of these are still not approved for the treatment of HF by the Food and Drug Administration (FDA), we aim to contribute to the existing data regarding these medications with the hope of seeing more research into the new domains of HF treatment.

## Review

Vericiguat

Vericiguat is a guanylate cyclase stimulator that works on the nitric oxide (NO), soluble guanylate cyclase (sGC), and cyclic guanosine monophosphate (cGMP) pathway, also known as the NO-sGC-cGMP pathway [3,4]. It enhances the cGMP pathway by stimulating sGC [3,5]. Its mechanism of action involves binding to a site that is independent of NO, and that in turn sensitizes sGC to endogenous NO by stabilizing NO binding to the binding site [3,5]. Vericiguat was preceded by riociguat, the first accepted sGC stimulator [3]. Currently, it is marketed in the United States under the brand name Verquvo [4]. It is indicated for symptomatic adults with chronic HF and ejection fraction (EF) <45% following hospitalization or the need for outpatient administration of intravenous (IV) diuretics [4].

Four clinical trials of vericiguat have concluded that it is as safe as a placebo, as summarized in Table 1. There were no adverse effects noted. No direct positive effects were noted on hospitalization periods and mortality [3].

**Table 1:** A summary of results of vericiguat trials HF: heart failure; HFpEF: heart failure with preserved ejection fraction; LVEF: left ventricular ejection fraction; NT-pro BNP: N-terminal pro-brain natriuretic peptide; sGC: soluble guanylate cyclase; EF: ejection fraction.

Name	Type	Aim(s)	Results
Vericiguat in patients with worsening chronic heart failure and preserved ejection fraction: results of the soluble guanylate cyclase stimulator in heart failure patients with preserved EF (SOCRATES-PRESERVED) study [6]	Phase 2b dose-finding study	To determine tolerance and optimum dose of vericiguat in patients with chronic HF and HFpEF.	Vericiguat was well-tolerated and demonstrated significant improvements in the quality of life of patients with HFpEF.
Effect of vericiguat, a soluble guanylate cyclase stimulator, on natriuretic peptide levels in patients with worsening chronic heart failure and reduced ejection fraction: the SOCRATES-REDUCED randomized trial [7]	Dose-finding phase 2 study	To determine optimal dosing and tolerability of vericiguat in patients with worsening chronic HF and reduced LVEF.	Compared with placebo, vericiguat was well-tolerated but did not have a significant effect on NT-pro BNP level.
Evaluate the efficacy and safety of the oral sGC stimulator vericiguat to improve physical functioning in daily living activities of patients with heart failure and preserved ejection fraction (VITALITY-HFpEF) [8]	Phase 2b study	To evaluate vericiguat (compared with placebo) in patients with HFpEF by assessing its efficacy using the physical limitation score of the Kansas City Cardiomyopathy Questionnaire.	In patients with HFpEF and recent decompensation, 24-week treatment with vericiguat (at 15 mg/day or 10 mg/day dosages) compared with placebo did not improve the physical limitation score of the Kansas City Cardiomyopathy Questionnaire.
Vericiguat in patients with heart failure and reduced ejection fraction (VICTORIA) [9]	Phase 3	To evaluate the efficacy and safety of vericiguat in patients with reduced EF and chronic HF with recent decompensation.	Vericiguat demonstrated no advantage over placebo with EF improvement in patients with EF ≥ 40%. Both groups of patients experienced similar progression. Treatment with vericiguat in addition to pre-existing standard care resulted in a 10% relative reduction in the primary composite outcome of death from cardiovascular causes or first hospitalization for HF.

A systematic review demonstrated that sGC stimulators such as vericiguat are safe and efficacious, and they reduce HF-associated hospitalization and mortality. However, several other meta-analyses revealed that vericiguat still showed lesser efficacy when compared with conventional medications such as beta blockers and sodium-glucose co-transporter-2 inhibitors (SGLT-2i) in patients with HF [3]. Pharmacologically, vericiguat was shown to reduce coronary perfusion pressure, along with improving cGMP levels in vascular smooth muscle cells. Hence, it has been hypothesized that suboptimal effects of vericiguat have been due to their effects on non-target cells [3,4].

Omecamtiv mecarbil

Omecamtiv mecarbil is an inotropic agent that acts as a myosin activator specific for cardiac cells [10]. Pre-existing inotropic agents that function by increasing intracellular calcium have displayed varying patient tolerance and adverse effects due to increased use of oxygen. Hence, omecamtiv mecarbil was developed as a safer alternative [10].

The mechanism of action of omecamtiv mecarbil includes binding to the catalytic domain of myosin, causing the adenosine triphosphate (ATP) equilibrium to move toward adenosine diphosphate (ADP-P) during systole. This, in turn, leads to an increased availability of myosin heads to bind to actin filaments. The end result is increased stroke power without an increase in cytoplasmic calcium or adverse effects of higher oxygen usage [10,11]. Clinical trials have shown that omecamtiv mecarbil is safe, well tolerated, and efficacious, as summarized in Table 2.

**Table 2:** A summary of omecamtiv mecarbil trials HF: heart failure; SET: systolic ejection time; NT-pro BNP: N-terminal pro-brain natriuretic peptide.

Name	Type	Aim(s)	Result
Acute treatment with omecamtiv mecarbil to increase contractility in acute heart failure: the ATOMIC-AHF study [12]	Phase 2 prospective trial	To compare placebo and omecamtiv mecarbil in patients with acute HF.	Omecamtiv mecarbil did not match the predicted dyspnea relief, except in the higher administration group. Omecamtiv mecarbil was well tolerated and increased SET.
Chronic oral study of myosin activation to increase contractility in heart failure (COSMIC-HF): a phase 2, pharmacokinetic, randomised, placebo-controlled trial [13]	Phase 2 trial	To demonstrate pharmacokinetics and optimal oral dosage of omecamtiv mecarbil in the treatment of chronic HF.	The results demonstrated the safety and tolerability of omecamtiv mecarbil. It also led to an increased SET and stroke volume but decreased the heart rate, NT-pro BNP level, and left ventricular volume.
Multicenter study to assess the efficacy and safety of omecamtiv mecarbil on mortality and morbidity in subjects with chronic heart failure with reduced ejection fraction (GALACTIC-HF) [14]	Phase 3 clinical trial	To measure the time of the first HF event or cardiovascular death during omecamtiv mecarbil therapy.	Omecamtiv mecarbil was well tolerated and safe in patients with severe HF. For cardiovascular deaths, omecamtiv mecarbil provided no reduction in mortality.

Ularitide

Ularitide is the synthetic form of urodilatin, which is a human natriuretic peptide that is produced in the distal renal tubule cells in response to increased blood pressure [10,15]. It binds to natriuretic peptide receptor-A (NPR-A), which is present in the heart, kidney, and vascular smooth muscle along with other organs, and it activates the intracellular guanylate cyclase (GC) domain of the receptor, also called particulate guanylate cyclase (pGC). pGC acts as a receptor for natriuretic peptides and leads to the formation of cGMP [15]. cGMP acts through several pathways, one of which is the cGMP-dependent protein kinase pathway. This leads to effects such as natriuresis and diuresis, vasodilation and bronchodilation, and inhibition of the renin-angiotensin-aldosterone system (RAAS) [10,15]. Findings from clinical trials of urodilatin and ularitide are summarized in Table 3.

**Table 3:** A summary of ularitide trials HF: heart failure; PCWP: pulmonary capillary wedge pressure; SVR: systemic vascular resistance; CHF: congestive heart failure; ANP-(95-126): atrial natriuretic peptide (95-126); SBP: systolic blood pressure; MAP: mean arterial pressure; CVP: central venous pressure; HR: heart rate; RAP: right atrial pressure; CI: cardiac index; cGMP: cyclic guanosine monophosphate.

Name	Type	Aim(s)	Results
Haemodynamic and renal effects of urodilatin in healthy volunteers [16]	Phase 1 study	To study hemodynamic and renal effects of synthetic urodilatin in a total of 18 healthy male volunteers.	Effects noted were an increase in heart rate, cardiac index as well as plasma cGMP levels (dose-dependent effect). PCWP and SVR were both noted to reduce. Diuresis and natriuresis were also noted.
Haemodynamic and renal effects of urodilatin bolus injections in patients with congestive heart failure [17]	Phase 1 study	To compare hemodynamic and renal effects of synthetic urodilatin with those of urodilatin in CHF.	Bolus injections of urodilatin were noted to produce positive hemodynamic effects in CHF patients. An increase in the average stroke volume index and a decrease in PCWP were reported respectively.
Efficacy of prolonged infusion of urodilatin [ANP-(95-126)] in patients with congestive heart failure [18]	Phase 1	To evaluate its therapeutic potentials in CHF, we investigated the efficacy of a prolonged infusion of urodilatin (15 ng/kg/min for 10 hours) in 12 patients with CHF (New York Heart Association functional classes II and III) in a randomized, double-blind, placebo-controlled study.	Factors noted to reduce were SBP, MAP, and CVP, and the factor noted to increase was HR (compared with the placebo group).
SIRIUS (Safety and efficacy of an Intravenous placebo-controlled Randomized Infusion of Ularitide in a prospective double-blind Study in patients with symptomatic, decompensated chronic heart failure): Effects of the renal natriuretic peptide urodilatin (ularitide) in patients with decompensated chronic heart failure [19,20]	Phase IIa	To study 24 acute decompensated HF patients requiring hospitalization and monitoring via right heart catheterization.	An increase in PCWP was noted with a decrease in RAP.
SIRIUS: Hemodynamic and clinical effects of ularitide in decompensated heart failure [19,20]	Phase IIb	To assess the efficacy and safety of ularitide in treating patients with decompensated HF.	A significant reduction in PCWP was noted with a significant improvement in shortness of breath. A reduction in SVR as well as an increase in CI was noted. No adverse effects were noted on the renal function of decompensated HF patients.
TRUE-AHF (Trial of Ularitide's Efficacy and safety in patients with Acute Heart Failure) [20]	Phase III	To evaluate the effects of ularitide on clinical status and 180-day mortality outcomes in acute HF.	Ularitide was concluded to have beneficial hemodynamic effects with a reduction in cardiac wall stress along with a reduction in SBP.

Serelaxin

Serelaxin is the synthetic recombinant form of relaxin, which is part of the relaxin family of peptides [10]. Relaxin binds to G protein-coupled receptors (GPCRs) present in the heart, lung, and kidney. Through GPCRs, relaxin leads to the stimulation of NO and vascular endothelial growth factor (VEGF) production and inhibition of vasoconstrictors. This, in turn, leads to increased cardiac output, reduced blood pressure, and systemic vascular resistance [10]. Based on clinical trials, serelaxin has been reported to be safe, and well tolerated, with a reduction in systolic blood pressure reported as well, as summarized in Table 4. In some trials, a reduction in hospital admissions as well as mortality rates was also reported.

**Table 4:** A summary of serelaxin trials HF: heart failure; GFR: glomerular filtration rate; PCWP: pulmonary capillary wedge pressure; NT-pro BNP: N-terminal pro-brain natriuretic peptide.

Name	Type	Aim(s)	Results
Influence of recombinant human relaxin on renal hemodynamics in healthy volunteers [21]	Phase 1	To assess the safety and tolerability of serelaxin and to demonstrate its beneficial effects.	Diuresis was increased with no significant change in GFR.
Intravenous recombinant human relaxin in compensated heart failure: a safety, tolerability, and pharmacodynamic trial [22]	Phase 1		Relaxin demonstrated no adverse effects. Hemodynamic effects noted were in line with vasodilation (i.e., increased cardiac index, decreased PCWP, and decreased circulating NT-pro BNP).
Pre-RELAX-AHF: Relaxin for the treatment of patients with acute heart failure [23]	Phase 2b study	To assess whether intravenous relaxin should be pursued in larger studies of acute HF, to identify an optimum dose, and to help assess endpoint selection and power calculations.	Serelaxin infusion showed a significant amount of dyspnea improvement. Another effect noted was a reduction in cardiovascular death or readmission.
RELAX-AHF: Serelaxin, recombinant human relaxin-2, for treatment of acute heart failure [24]	Phase 3 study	After the above-mentioned pre-RELAX-AHF trial, the primary endpoints evaluating dyspnea improvement were changed from baseline in the visual analog scale area under the curve to day five and the proportion of patients with moderate or marked dyspnea improvement measured by the Likert scale during the first 24 hours, with both being analyzed by intention to treat.	A significant relief in dyspnea was reported with an improvement in other clinical outcomes, but no effect on readmission to the hospital was noted. Serelaxin treatment was well tolerated and safe.
The efficacy, safety, and tolerability of additional serelaxin administration to standard therapy in Asian patients with acute heart failure: the RELAX-AHF-ASIA trial [25]	Phase 3	To evaluate the effects of serelaxin in Asian patients.	Only exploratory analysis for primary and secondary endpoints was performed (due to premature termination of this trial).
RELAX-AHF-2: Effects of serelaxin in patients with acute heart failure [26]	Phase 3	To confirm the beneficial effects of serelaxin on 180-day cardiovascular death and worsening HF by day five, with secondary endpoints including 180-day all-cause mortality, cardiovascular death or re-hospitalization, and length of hospitalization.	There was no difference in cardiovascular mortality at 180 days. There was a reduction of worsening HF noted but it was not statistically significant.

## Conclusions

As the prevalence of HF continues to increase globally, the need for alternative medications keeps increasing. Although we have seen better outcomes with the medications mentioned in this review, three of them (omecamtiv mecarbil, ularitide, and serelaxin) are still not approved for clinical use for HF patients.

In conclusion, further studies are required to compare the above-mentioned medications with the existing standard of care for HF patients. It remains to be noted if vericiguat, omecamtiv mecarbil, ularitide, and serelaxin potentially provide a better and more efficacious alternative for HF patients who might not show adequate improvement with the existing medications, or for HF patients who might not be able to tolerate conventional medications.
